# Supplementary material for: Minerals in the pre-settled coral Stylophora pistillata crystallize via protein and ion changes
Source: Nat Commun. 2018 May 14;9:1880. doi: 10.1038/s41467-018-04285-7 (PMC5951882; doi:10.1038/s41467-018-04285-7)
Supplement: Supplementary file 1 — Supplementary Information [file 41467_2018_4285_MOESM1_ESM.pdf]

Supplementary Information for

Minerals in the Pre-Settled Coral *Stylophora pistillata* Crystallize via Protein and Ion Changes

Anat Akiva,[1#] Maayan Neder,[2,3] Keren Kahil,[1] Rotem Gavriel,[4] Iddo Pinkas,[5] Gil Goobes,[4*] Tali Mass[2*]

* Corresponding authors: talimass@univ.haifa.ac.il, gil.goobes@biu.ac.il

Supplementary Note

The DE ^{13}C NMR spectra of the pre-settled planula and primary polyp, shown in Figs. 4B and 4C, are further analyzed by line deconvolution. In Supplementary Figures 4 and 5, we show the deconvolution of the carbonate/carbonyl region of the planula and polyp, respectively, along with tables summarizing the constituent lines, in terms of chemical shift, line width, line shape and relative intensity. The mineral lines are shown in bold-face. The aragonite line normalized to the weight of the sample is more intense in the primary polyp, indicating that more mineral is found in the organism at this stage. Its full width at half maximum (FWHM) also decreases from 2.3 to 1.3 ppm, reflecting an increase in the order inside the aragonite crystallites post-settlement.

The proton-enhanced ^{13}C CP spectra (Fig. 4D) only show the mineral-phase carbonates with protons in their vicinity. These are typically mineral layers on crystal faces and hydrated or dry $CaCO_3$ phases with variable degrees of disorder. A zoom-in on the carbonate region (Fig. 4E) shows the broad resonance of the mineral phase overlapping with the band of the carbonyl carbons from the backbone of various proteins. Detailed deconvolutions of these peaks and tables summarizing all peak parameters are given in Supplementary Figures 6 and 7, with the mineral lines shown in bold-face. The carbonate peaks at 169.3 ppm (FWHM 4.8 ppm) and 170.7 ppm (FWHM 4.5 ppm) in the pre-settled planulae and primary polyps, respectively, are readily ascribed to highly disordered $CaCO_3$, referred to as ACC.

The 2D ^{1}H-^{13}C HETCOR experiment produces a two-dimensional map of the proton-to-carbon magnetization transfer patterns in each sample, with the vertical axis representing proton chemical shift and the horizontal axis carbon chemical shift. The spectra for the two developmental stages are shown Figs. 5B and S8. While the carbohydrate region at 70–112

ppm in the two stages is identical, differences are seen in the protein carbon resonances. Unique peaks are observed in the polyp at 34 and 35.5 ppm, attributed to Val or Lys Cβ, at 50 ppm, attributed to Ala Cα and at 177–179 ppm of the Cδ carboxylate of Asp. Unique peaks are also observed in the planulae at 22 and 43 ppm, attributed to Leu and Arg Cδ carbons, and at 68 ppm, attributed to either Thr Cβ carbons or some other biomolecule. The prominence of the Asp, Val, Lys and Ala carbons, abundant in CARP1, CARP3 and CARP4 in the polyp spectrum, is consistent with the increased expression of these proteins in the later stage of the coral. The prominence of the Leu and Arg carbons, abundant in CARP2 in the planula spectrum, is consistent with its over-expression in the early developmental stage. Additional Cα/Cβ carbons in the primary polyp cover shifts between 58 and 66 ppm owing to the larger number of proteins expressed at this stage and their larger span of different prevalent amino acids. The narrower Cα/Cβ carbon band of shifts between 62 and 64 ppm in the pre-settled form is in accordance with the dominant expression of CARP2, which has a narrower distribution of prevalent amino acids in its sequence (see Supplementary Table 2). Some chemical-shift deviations were allowed in the above analysis, considering the possible changes within the mineral environment relative to the solution. The glutamate amino acid is abundant in all CARPs and, therefore, its level cannot be used as an indicator of differential expression in the two stages. Moreover, its carboxylate residue is not observed in Supplementary Figures 8.

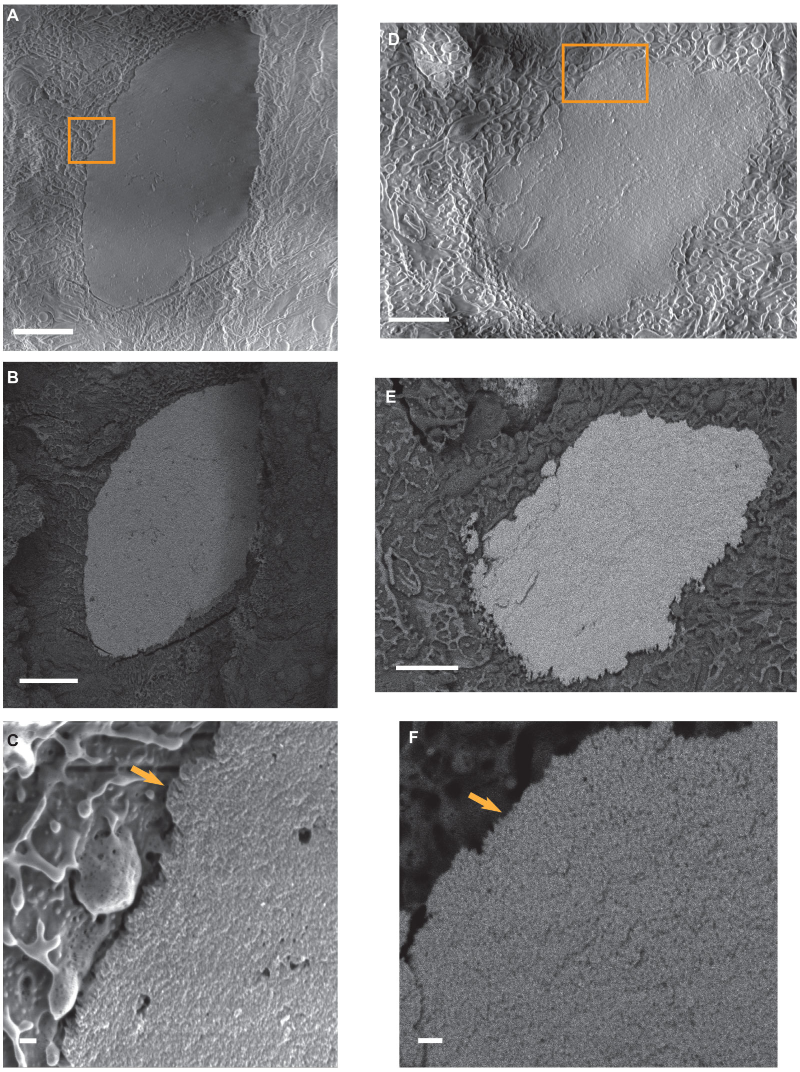

Supplementary Figure 1: Mineral deposits at the pre-settled larval stage, detected by cryo-SEM. (A-F) Several different extracellular mineral deposits. (A, D) SE image of a mineral deposit. The mineral is ~10 µm or larger. (B, E) BSE image of the same region as in (A). (C, F) Higher magnification of the region delimited by an orange box in (A, D). The granular structure is visible at the interface between the mineral and the organic matrix (orange arrows). A custom mask Fourier filter was used to remove the charging effects in A and D. Scale bars: A, B, 2 µm; C, F, 200 nm; D, E, 5 µm.

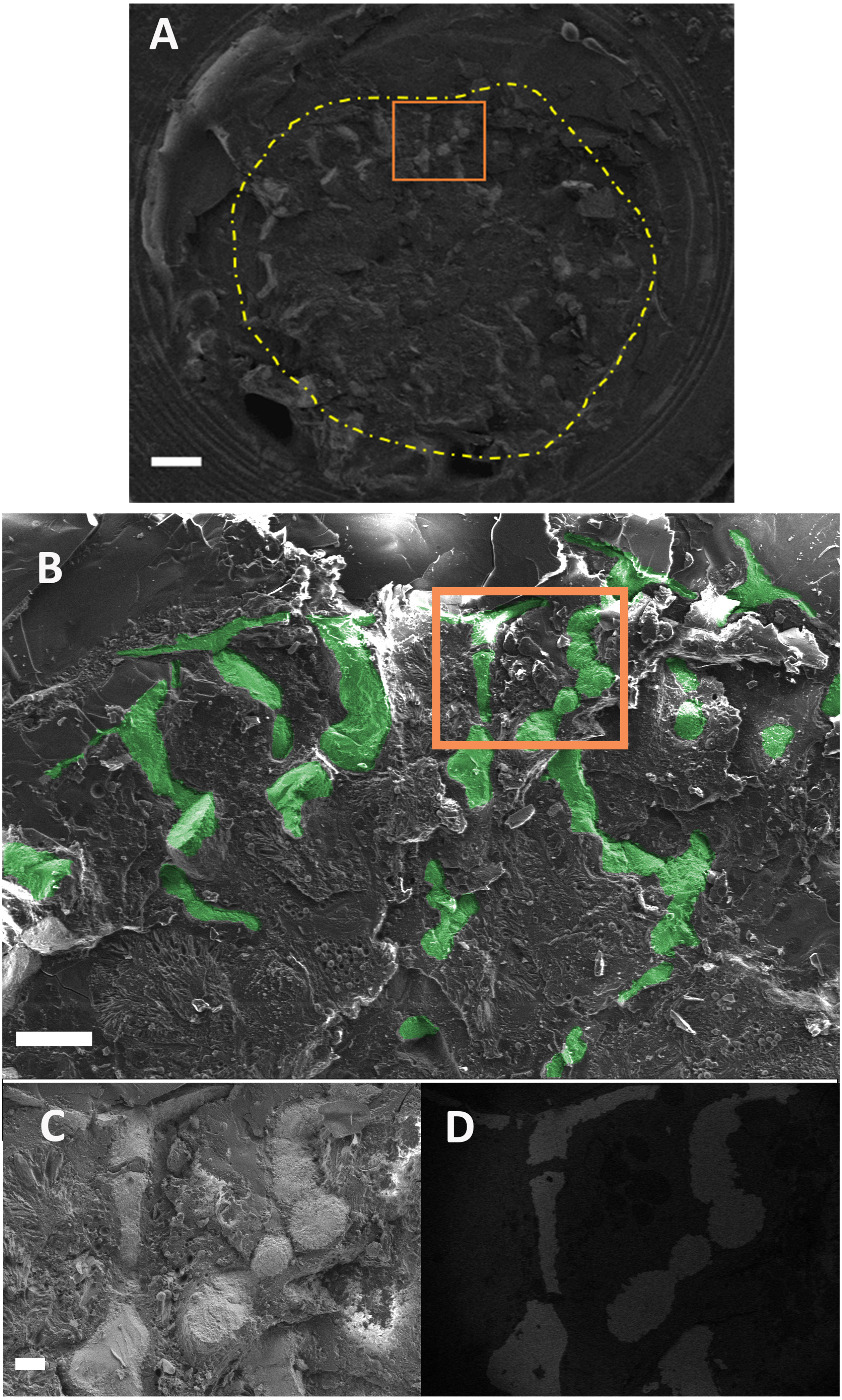
A
B
C
D

Supplementary Figure 2: Cryo-SEM of freeze-fractured primary polyp (A-D). (A) Low magnification cryo-SEM image in SE mode of a primary settled polyp after high pressure freezing and freeze fracture; (B) SEM image of the region delimited by an orange box, a well-developed septa of primary polyp after settlement. The primary and secondary septa are shaded in green for clarity. (C) SEM image of the region delimited by an orange box. (D) BSE image of the same region as in (C). Scale bars: A, 200 µm; B, 100 µm; C, D, 20 µm.

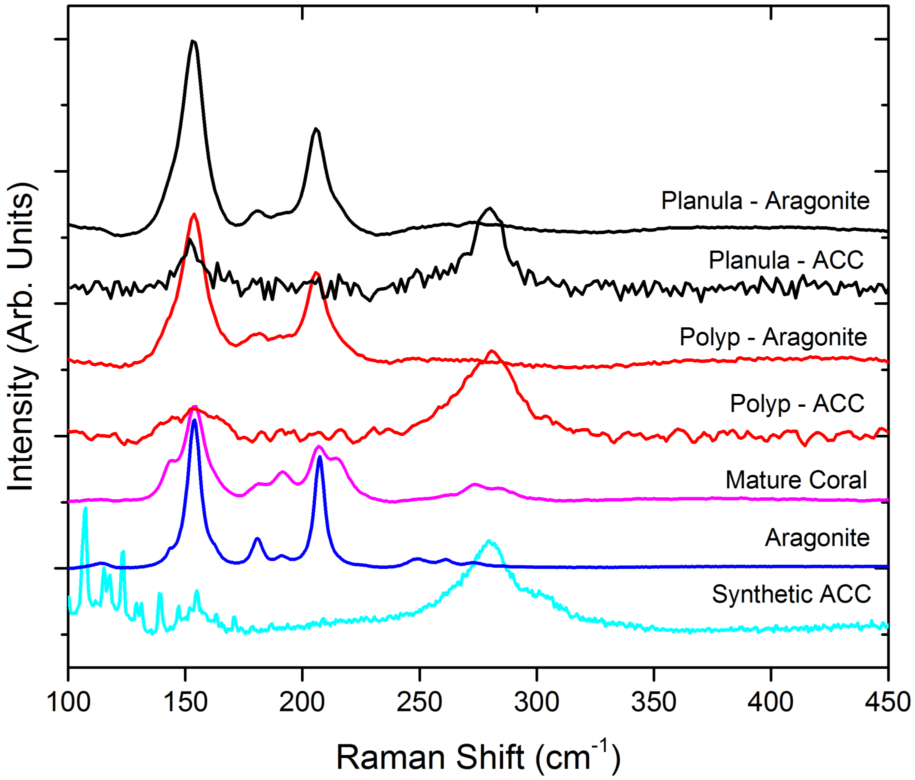

Supplementary Figure 3: Raman spectra focusing on the low wavenumber region (slow vibrations of the unit cell) of the materials. The low-intensity broad peak that is detected at ~270 cm^{-1} in the different coral samples is characteristic of ACC.

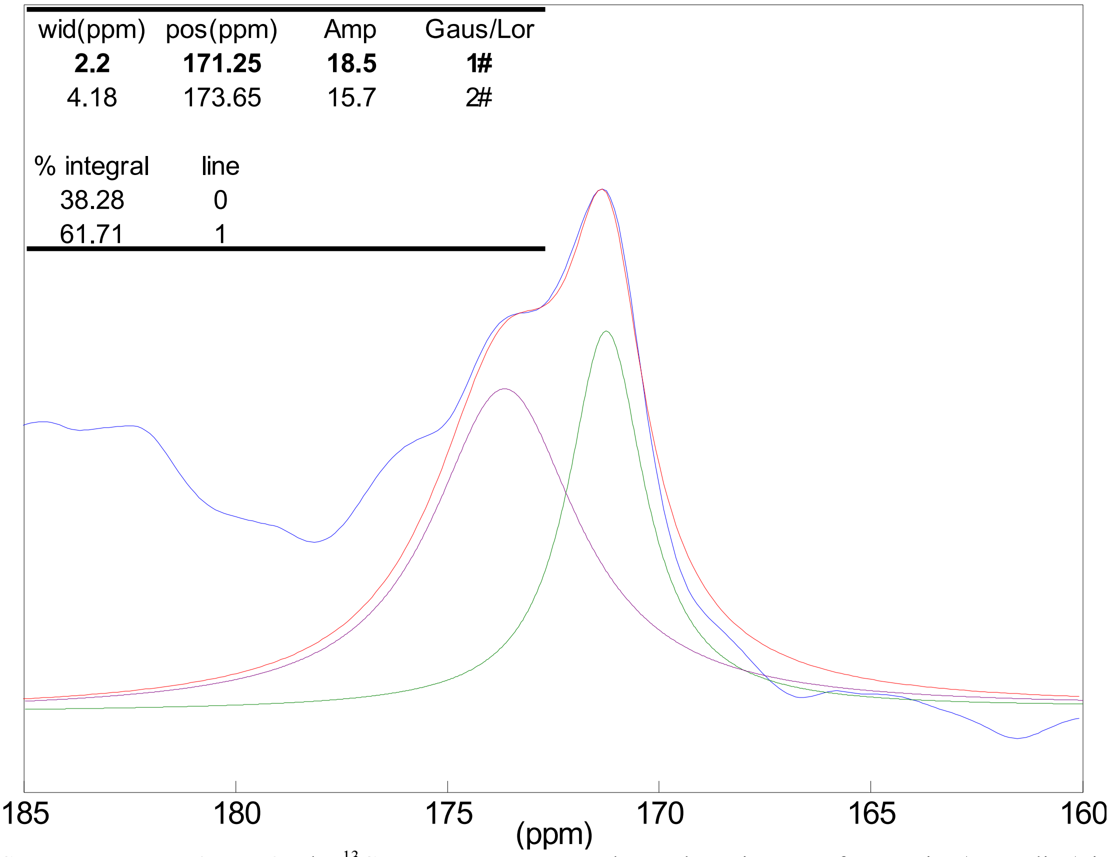

Supplementary Figure 4: The ^{13}C DE NMR spectrum shows the existence of aragonite (green line) in the pre-settled planulae. The aragonite line has a width of 2.2 ppm (FWHM) indicating that this phase is less ordered than in the primary polyp as can be seen by the linewidth difference. Deconvolution of the peaks in the carbonate/carbonyl region of the ^{13}C DE spectrum of the ^{13}C-enriched pre-settled planulae. Experimental spectrum is in blue, sum of simulated lines in red and individual peaks of carbonyl in purple and carbonate in green. Inset table lists the lines fitted, and the carbonate line is in bold. In the inset table, the line number in the integral calculation is skewed by 1 relative to the number in the upper rows, i.e.: line 0 -> 1, 1-> 2.

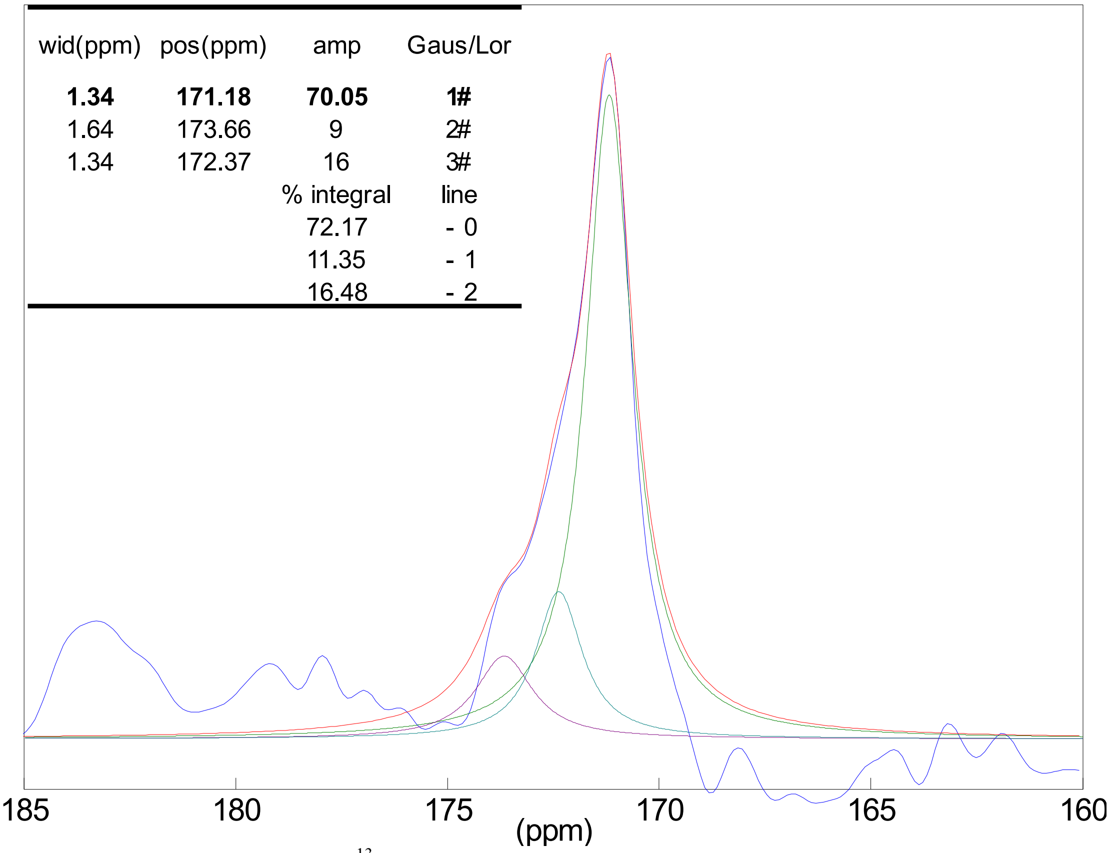

Supplementary Figure 5: The ^{13}C DE NMR spectrum shows the existence of aragonite (green line) in the primary polyp in higher percentage than in the pre-settled planulae. Here the aragonite line is much narrower measuring 1.34 ppm (FWHM), indicating increased order in the crystallites also called "maturation"[1]. Deconvolution of the peaks in the carbonate/carbonyl region of the ^{13}C DE spectrum of the ^{13}C-enriched post-settlement polyps. Experimental spectrum is in blue, sum of simulated lines in red and individual peaks of carbonyl in purple and in cyan and carbonate in green. Inset table lists the lines fitted, and the carbonate line is in bold. In the inset table, the line number in the integral calculation is skewed by 1 relative to the number in the upper rows, i.e.: line 0 -> 1, 1-> 2, 2-> 3.

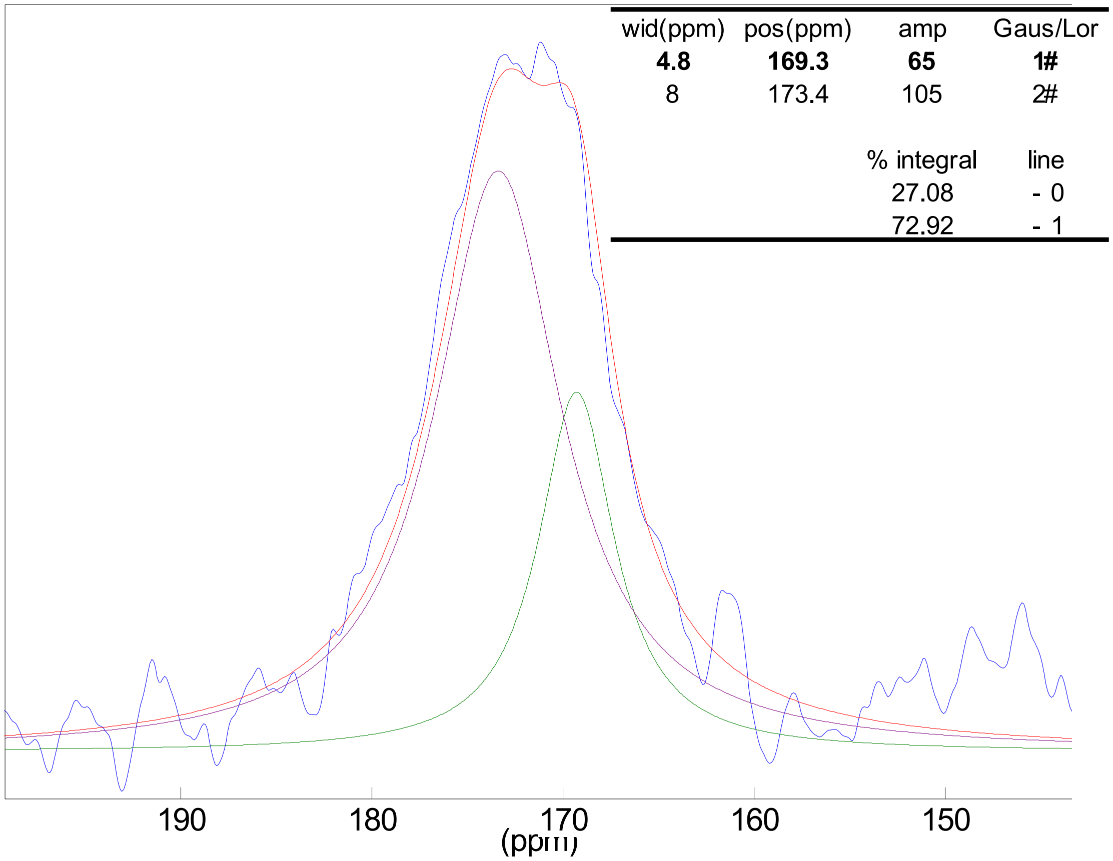

Supplementary Figure 6: The ^{13}C CP NMR spectrum shows the existence of an amorphous calcium carbonate phase (green line) in the pre-settled planula. Deconvolution of the peaks in the carbonate/carbonyl region of the ^{13}C CP spectrum of the ^{13}C-enriched pre-settled planulae. Experimental spectrum is in blue, sum of simulated lines in red and individual peaks of carbonyl in purple and carbonate in green. The table shows the spectral properties of each line in the fit. Inset table lists the lines fitted, and the carbonate line is in bold. In the inset table, the line number in the integral calculation is skewed by 1 relative to the number in the upper rows, i.e.: line 0 -> 1, 1-> 2.

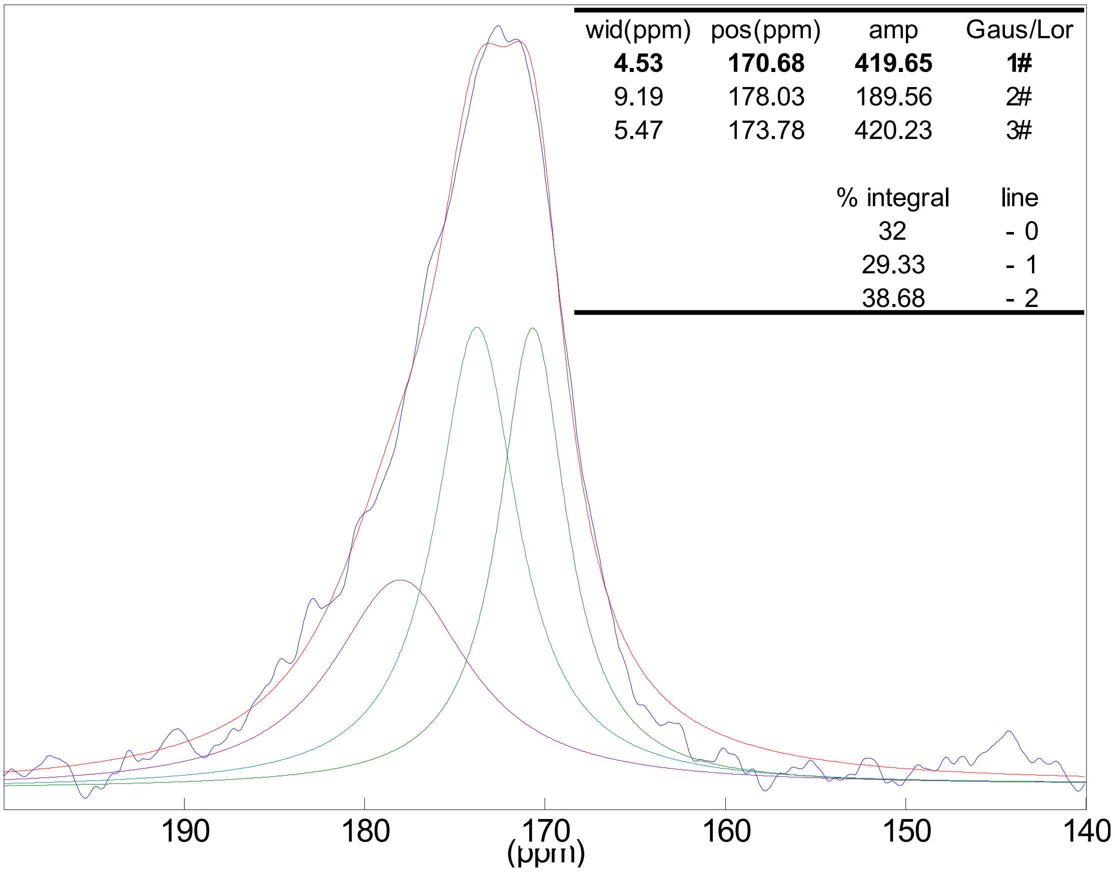

Supplementary Figure 7: The ^{13}C CP NMR spectrum shows the existence of a disordered aragonite phase (green line) in the primary polyp. The mineral line appears at 170.68 ppm in the polyp and at 169.3 ppm in the planula marking a major change in the identity of the mineral from amorphous to disordered aragonite as judged by the small decrease in line width from 4.8 to 4.53 ppm with metamorphosis. Deconvolution of the peaks in the carbonate/carbonyl region of the ^{13}C CP spectrum of the ^{13}C-enriched post-settlement polyps. Experimental spectrum is in blue, sum of simulated lines in red and individual peaks of carboxylate in purple, carbonyl in cyan and carbonate in green. The table shows the spectral properties of each line in the fit. Inset table lists the lines fitted, and the carbonate line is in bold. In the inset table, the line number in the integral calculation is skewed by 1 relative to the number in the upper rows, i.e.: line 0 -> 1, 1-> 2, 2-> 3. The asymmetry of the carbonate/carbonyl line required the addition of a line at 178 ppm attributed to carboxylate sidechains typically observed in aspartic acid residues.

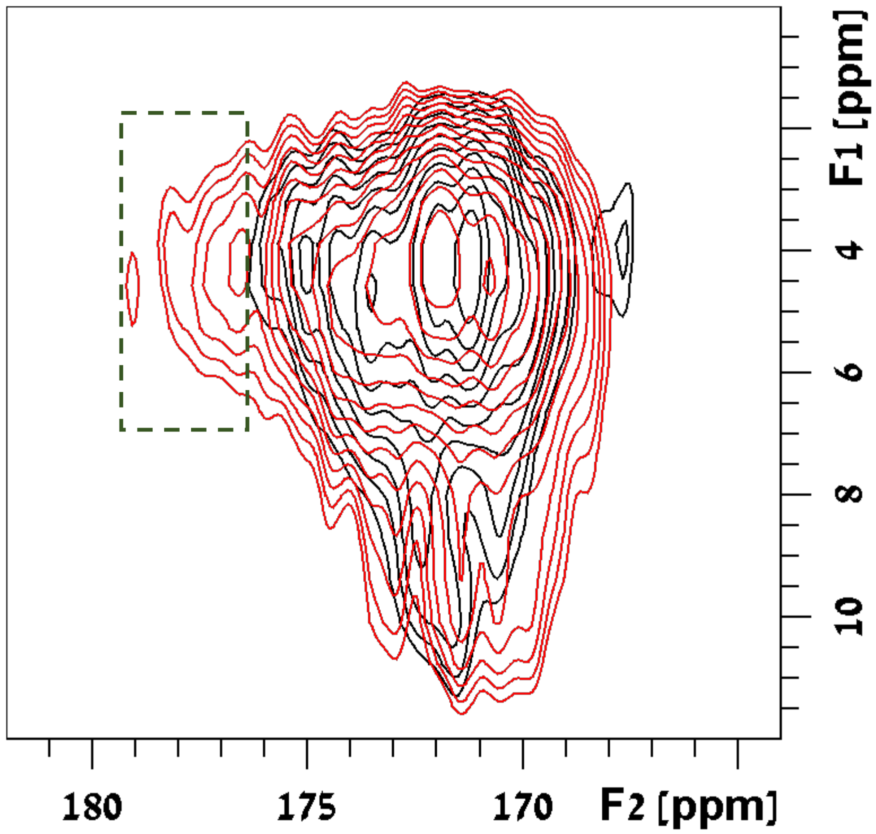

Supplementary Figure 8: Carbonate region in the 2D ^{1}H-^{13}C HETCOR spectra of pre- (black) and post-settlement (red) polyps. Spectra were recorded using a contact time of 2 ms, a recycle delay of 1 s and 4,000 scans. The aspartate cross peak region, observed only in the polyp, is delimited by a dashed square.

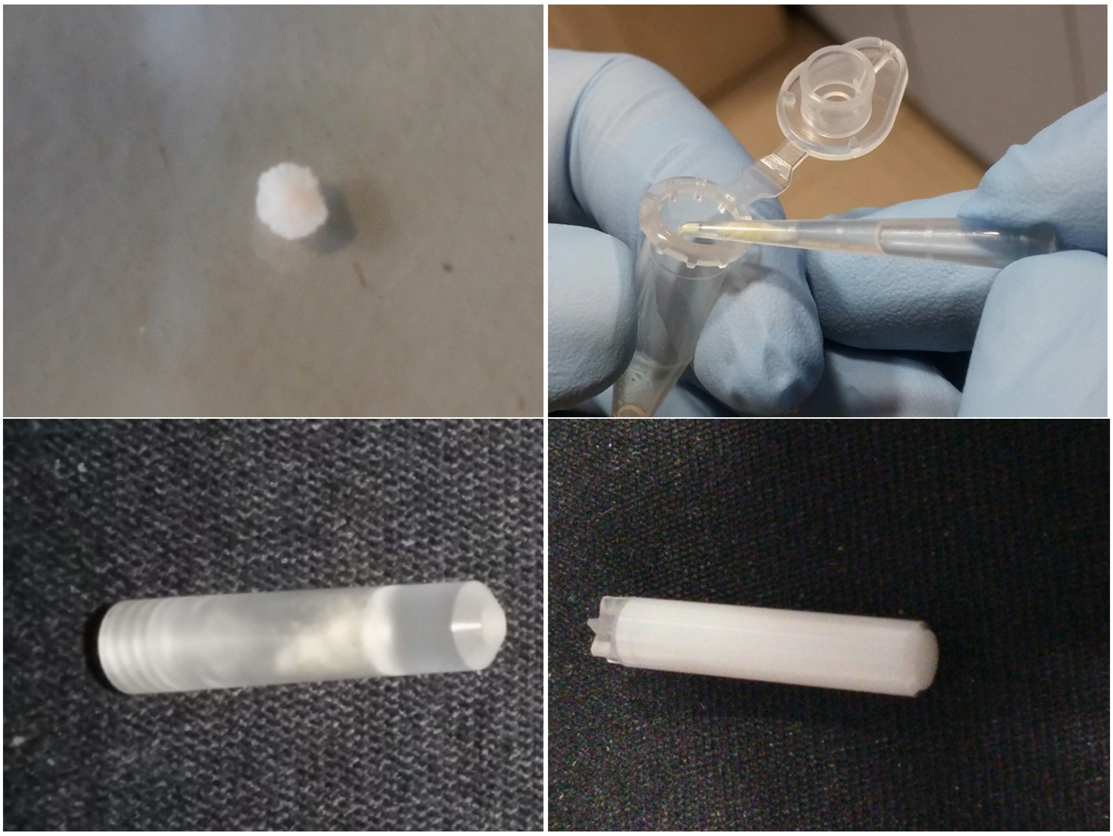

Supplementary Figure 9: The planulae were collected from the solution by spin-down and the settled polyps were scraped off the petri dish using tweezers. **(a)** An example of settled polyp where mineral formation gives rise to an opaque image. **(b)** Collected organisms are washed in D_2O and then **(c)** lyophilized, formed, transferred into the Kel-F insert and **(d)** packed in a MAS rotor.

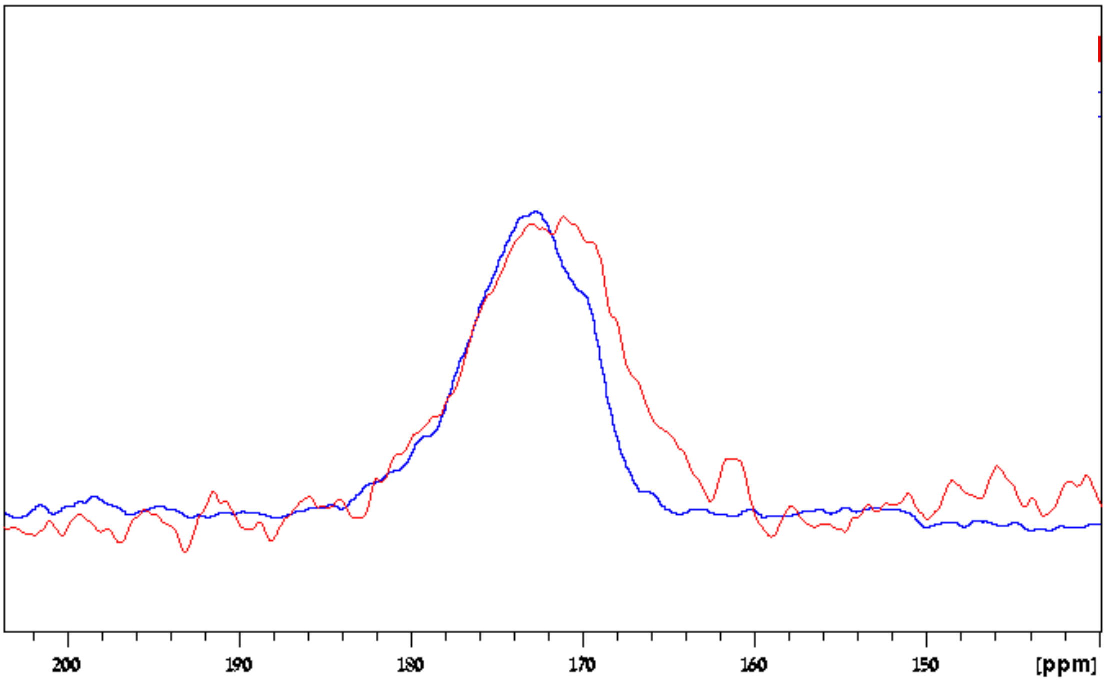

Supplementary Figure 10: Carbonate/carbonyl region of the ^{13}C CP spectrum of the ^{13}C-enriched pre-settled planulae, fresh packed (red) and two weeks later (blue).

Supplementary Table 1: The complete assignment of the vibrational modes that were acquired by micro-Raman. The assignments are following the paper of De La Pierre et al.[1] listing the symmetry of the crystal vibrational mode.

Aragonite				Mature coral				primary polyp				pre-settled planula			
Raman Shift (cm^{-1})	S.G	#	FWHM	Raman Shift (cm^{-1})	S.G	#	FWHM	Raman Shift (cm^{-1})	S.G	#	FWHM	Raman Shift (cm^{-1})	S.G	#	FWHM
153,8	B1g	11	6,9	154,1	B1g	11	9,7	153,3	B1g	11	13,6	153,2	B1g	11	11,2
180,7	B2g	16	4,5	180,9	B2g	16	6,8	175,8	B2g	16	83	183,2	B2g	16	15,8
207,4	B2g	17	5,4	207	B2g	17	8,3	206,4	B2g	17	11,7	206,2	B2g	17	9,7
701,7	B3g	29	1,9	707,1	Ag	6	4,3	706,9	Ag	6	6,2	706,8	Ag	6	6,1
1085,9	Ag	8	1,4	1086,8	Ag	8	4	1086,9	Ag	8	3,7	1086,2	Ag	8	5
144	Ag	1	3,8	191,2	B3g	27	6,9	193,6	B3g	27	76	192,4	B3g	27	34
188,6	B3g	27	12	215,5	Ag	4	8	263,5	B2g	19	17,6	260,4	B2g	19	15,7
249,1	B2g	18	8,5	273,5	B3g	28	10	271,9	B3g	28	?	273,3	B3g	28	28,5
261	B2g	19	7,6	284,9	Ag	5	10,1	702,6	B3g	29	3,9	280,9	Ag	5	20,6
273,6	B3g	28	11	702,8	B3g	29	3,6	730	B2g	21	70	702,2	B3g	29	5,6
163,1	Ag	2	4,1	854,8	Ag	7	3,9					718,2	B2g	21	11,5
113,6	B1g	10	1,3	1463,5	B1g	15	19,6								
706,5	Ag	6	2,1												
717,2	B2g	21	1,9												
853,7	Ag	7	2,7												
1462,4	B1g	15	4,3												

Supplementary Table 2: Abundance of the four most frequent amino acids in the sequences of CARPs 1–4 and STPCA2

CARP1	Asp (17.9%)	Glu (16.7%)	Lys (11.5%)	Leu (5.8%)
CARP2	Glu (28.5%)	Arg (12.1%)	Leu (9.5%)	Lys (7.8%)
CARP3	Asp (35.0%)	Glu (15.3%)	Ala (13.4%)	Ser (7.6%)
CARP4	Asp (21.8%)	Val (8.7%)	Lys (7.4%)	Phe (6.7%)
STPCA2	Lys (9.8%)	Leu (7.9%)	Gly (7.9%)	Ser (7.0%)

Supplementary Table 3: qPCR primer information used.

Gene	Sequence	Amplicon size (bp)	qPCR T_{anneal} (^{o}C)	Reference
**CARP1**	F-TCACCTTGAACACGACGCAT<br>R-TGGAATCGCTGCCTGATCTC	159	60	This work
**CARP2**	F-TTGTGGACAGGGAACTTCCTGA<br>R-CTTCGGAGATTCATGCTCC	174	60	This work
**CARP3**	F-ACGTTCCAGAAAGTGCCAATG<br>R-TTTTGTGTCAGCGGCAGCAT	131	60	This work
**CARP4**	F-TCATGAACCTGGGAGTGTCAGA<br>R-TTCTCCAGGTGAATCGTCATCG	121	60	This work
**Actin**	F-GTCGAGACGAAGGATCGCAT<br>R-ACGTTGCCATCCAAGCTGTA	140	60	This work
**CA**	F-AGGACTCCAAGAACTGCAAG<br>R-TTCCTGCTCAGCTTCACTTGG	97	60	Bertucci et al. 2100

Supplementary References

1 Von Euw, S. *et al.* Biological control of aragonite formation in stony corals. *Science* **356**, 933-938, doi:10.1126/science.aam6371 (2017).

2 Bertucci, A., Tambutté, S., Supuran, C., Allemand, D. & Zoccola, D. A new coral carbonic anhydrase in *Stylophora pistillata*. *Mar. Biotechnol.* **13**, 992-1002, doi:10.1007/s10126-011-9363-x (2011).
